# Fiber optic sensor based on ZnO nanowires decorated by Au nanoparticles for improved plasmonic biosensor

**DOI:** 10.1038/s41598-019-52056-1

**Published:** 2019-10-30

**Authors:** Hyeong-Min Kim, Jae-Hyoung Park, Seung-Ki Lee

**Affiliations:** Department of Electronics and Electrical Engineering, Dankook University, Yongin, 16890 South Korea

**Keywords:** Nanoscience and technology, Optics and photonics

## Abstract

Fiber-optic-based localized surface plasmon resonance (FO-LSPR) sensors with three-dimensional (3D) nanostructures have been developed. These sensors were fabricated using zinc oxide (ZnO) nanowires and gold nanoparticles (AuNPs) for highly sensitive plasmonic biosensing. The main achievements in the development of the biosensors include: (1) an extended sensing area, (2) light trapping effect by nanowires, and (3) a simple optical system based on an optical fiber. The 3D nanostructure was fabricated by growing the ZnO nanowires on the cross-section of optical fibers using hydrothermal synthesis and via immobilization of AuNPs on the nanowires. The proposed sensor outputted a linear response according to refractive index changes. The 3D FO-LSPR sensor exhibited an enhanced localized surface plasmon resonance response of 171% for bulk refractive index changes when compared to the two-dimensional (2D) FO-LSPR sensors where the AuNPs are fixed on optical fiber as a monolayer. In addition, the prostate-specific antigen known as a useful biomarker to diagnose prostate cancer was measured with various concentrations in 2D and 3D FO-LSPR sensors, and the limits of detection (LODs) were 2.06 and 0.51 pg/ml, respectively. When compared to the 2D nanostructure, the LOD of the sensor with 3D nanostructure was increased by 404%.

## Introduction

Localized surface plasmon resonance (LSPR)^1–3^, chiral plasmonic^4,5^, magneto plasmonic^6^, surface enhanced infrared adsorption^7,8^ biosensors using plasmonic nanoparticles with receptor that specifically binds to target biomolecules have attracted much interest because of their characteristics such as label-free and real-time measurements. In particular, LSPR is well known and studied a lot due to the well-established theory, simple optical system, and compactness of the device^9–12^. LSPR is generated by the interaction between incident light and collective oscillation of the free electrons on the surface of nanoparticles and has the property that can be observed in the wavelength of the visible region^13^. LSPR depends on the material^14,15^, shape^16^, size^17^, and dielectric environment^18,19^ of the noble metal nanoparticles. So it is possible to analyze the interaction of biomolecules on the particles surface by observing the changes of the LSPR intensity^20^, wavelength^21^, and phase^22^ depending on the refractive index of the dielectric medium at the metal-dielectric interface.

Despite the high sensitivity of plasmonic biosensors, the demand for a more sensitive LSPR sensor is not met, and many types of research are still underway. For example, studies that control the density and size of nanoparticles and extend the substrate are reported in order to achieve higher sensitivity^23–25^. However, these methods have limitations in increasing the ratio of nanoparticles per unit plane because nanoparticles are two-dimensionally arranged on the sensor surface, which is an obstacle to obtain the LSPR sensor with better sensitivity^26^. As a countermeasure to increase the number of nanoparticles in a defined area, the LSPR sensors having a three-dimensional (3D) arrangement of nanoparticles by combining nanowires and nanoparticles have been studied^27–29^. In the structure in which the nanowires and the nanoparticles are combined, the nanowires increase the surface area of the sensor to which the particles are to be immobilized and trap the incident light to the sensing region because of their forest-like structure^30^. As a result, enhanced interaction of incident light to plasmonic nanoparticles leads to improved sensitivity of the LSPR sensors^31^.

In this paper, a highly sensitive 3D nanostructure based on nanowires-nanoparticles composite is conjugated with an optical fiber platform. When an optical fiber is used as a substrate instead of general substrates such as glass and silicon wafer, there are the following advantages. Since light can be delivered and received through an optical fiber, the configuration of the optical set-up is relatively simple, the measurement system can be miniaturized, and the handling is easy. It is also independent of the effects of electromagnetic waves, guarantees a small signal loss and allows remote sensing. Due to these advantages, fiber-optic-based LSPR (FO-LSPR) sensors are recently considered as promising candidates for biosensor applications^32–34^. The combination of a highly sensitive 3D LSPR nanostructure based on the label-free real-time measurement and an optical fiber platform with miniaturization and portability characteristics will be considered as a feasible attempt for point of care.

Three-dimensional (3D) distribution of nanoparticles was facilitated by synthesizing zinc oxide (ZnO) nanowires on the surface of an optical fiber as supporting materials. Gold nanoparticles (AuNPs) were then immobilized on the nanowires. ZnO nanowires were selected because of their advantages including optical transparency, biocompatibility, ease of fabrication, and well-known synthesis method^35,36^. The ZnO nanowires increase the sensing area together with the 3D array of nanoparticles and reduce the loss of light by capturing optical signals from the fiber in the sensing area. In addition, the nanoparticles that are lifted from the optical fiber’s surface by the nanowires can improve the LSPR efficiency via the enhanced effect of electric field concentration according to the reduction of substrate effect^37^. To prove that the sensitivity is enhanced in the proposed sensor, this parameter was measured for a two-dimensional (2D) distribution of nanoparticles and for a 3D nanostructure. The optical properties were then compared between 2D and 3D FO-LSPR sensors. Each sensor was also utilized for the measurement of antibody-antigen reaction with various concentrations and the change of the output intensities were compared. The measured results confirm that the 3D distribution of nanoparticles yielded a higher sensitivity compared to the monodispersed nanoparticles on an optical fiber.

## Material and Methods

### Fabrication process

**Figure 1:**
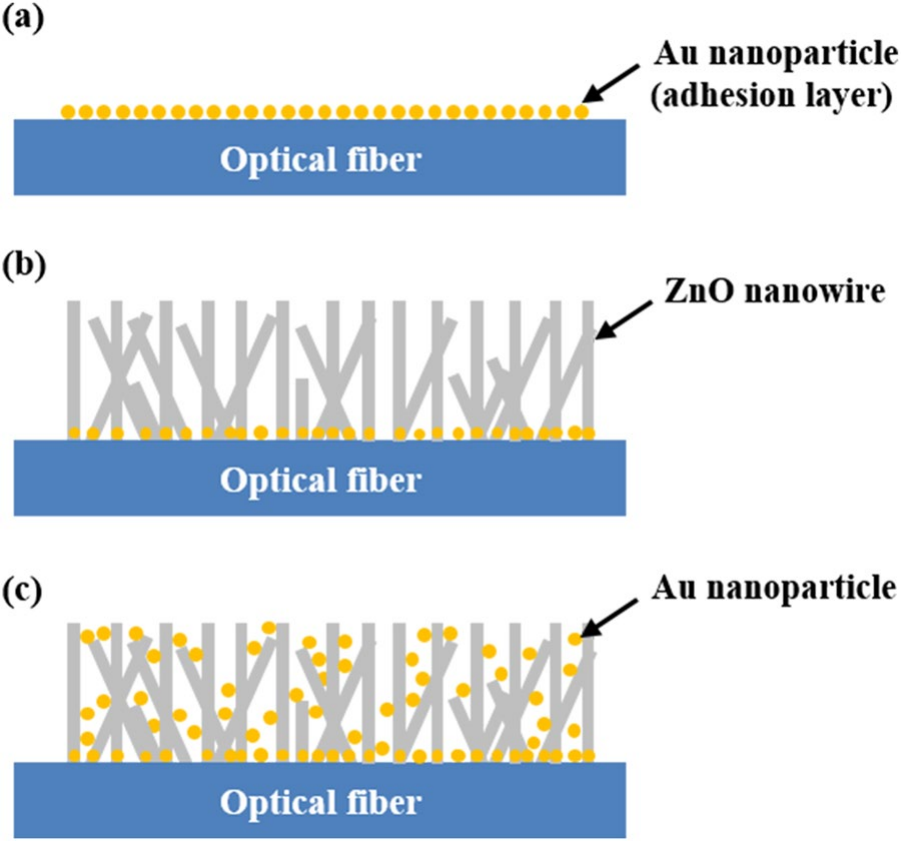
Fabrication process of ZnO nanowires and AuNPs composite structure: (**a**) fixation of AuNPs on optical fiber, (**b**) growth of ZnO nanowires, and (**c**) coating of AuNPs on ZnO nanowires.

Figure 1 describes the overall fabrication process for an FO-LSPR sensor with a 3D nanoparticle array and ZnO nanowire support. Several investigations have been performed regarding the requirement of a seed layer when ZnO nanowires are grown on a substrate^38,39^. Perpendicular and uniform ZnO nanowires can be fabricated in the presence of a seed layer. Although there are many available techniques used to form seed layers such as radio frequency magnetron sputtering deposition, pulsed laser deposition, sputtering, and thermal oxidation, the most simple and cost-effective method is to use a ZnO colloid solution^40,41^. However, ZnO nanoparticles in a colloid solution are difficult to introduce directly onto an optical fiber due to the small cross-sectional area of the fiber and the low adhesion force between the ZnO nanoparticles and the surface of the optical fiber.

Figure 1(a) shows the first step in the formation of an adhesion layer composed of AuNPs for the adsorption of ZnO nanoparticles as a seed layer on an optical fiber. The fabricated structure in this step was then used as a 2D FO-LSPR sensor for comparison with a 3D sensor. To fix the AuNPs onto the optical fiber, three processes were performed. Firstly, the flattened optical fiber (FG105LCA, Thorlabs, USA) was immersed in a mixed solution with a 1:3 ratio of hydrogen peroxide (34.5%, Samchun, South Korea) and sulfuric acid (95%, Daejung, South Korea) for 20 min to functionalize the hydroxyl groups on the cross-section of the fiber. Secondly, amine groups were formed on the optical fiber’s surface using 3-aminopropyldimethylethoxysilane (APMES, Gelest, USA) solution which has an ethoxy group to substitute for the hydroxyl group at one end and an amine group to adsorb the AuNPs at the other end. APMES was diluted to 5% with isopropyl alcohol (99.5%, Daejung) and the reaction time in solution was 60 min. Finally, the optical fiber was incubated for 30 min in an Au colloid aqueous solution that was prepared by mixing 250 µM Au(III) chloride trihydrate (99.9%, Sigma Aldrich, USA) and 35 mM sodium citrate dihydrate (99%, Daejung) in a volume ratio of 1:100. The negative charges on the surface of citrate-reduced AuNPs electrostatically bonded to the amine groups on the optical fiber’s surface^42^. The UV-Vis spectrum of prepared Au colloid aqueous solution was showed in Fig. S1.

In Fig. 1(b), ZnO nanowires are grown via hydrothermal synthesis^43^. To produce the ZnO colloid solution that was used as the seed layer, 2.5 mM zinc acetate dehydrate (99%, Junsei, Japan) alcohol solution was stirred in a hot bath at 65 °C for 2 h. The prepared solution was coated on an optical fiber and AuNPs were attached and dried using a hot plate ay 355 °C (HSD180, MTOPS, South Korea) for 1 min. This process was repeated 20 times to uniformly coat the seed particles onto the optical fiber. These particles were immobilized over the AuNPs because of the covalent bond between the hydroxyl groups on the ZnO nanoparticles and AuNPs^44^. After the sample was annealed at 355 °C for 30 min using a hot plate, ZnO nanowires were synthesized on the optical fiber in a 1:1 aqueous mixture of 10 mM zinc nitrate hexahydrate (98.0%, Samchun) and 10 mM hexamethylenetetramine (99.0%, Samchun). The growth condition was 105 °C in an oven for 2 h (TH-ME-025, Jeio Tech, South Korea). The length of the nanowires was set to a target of approximately 200 nm within a range in which the substrate effect is reduced and the plasmonic effect is not significantly degraded^45,46^. The grown ZnO nanowires were characterized by X-ray diffraction (Fig. S2).

To immobilize the AuNPs on a 3D support made of ZnO nanowires as shown in Fig. 1(c), the surface of the nanowires was initially treated using oxygen plasma (CUTE, Femto Science, South Korea) for 50 seconds to generate hydroxyl groups at a power of 60 W and a pressure of 0.8 mTorr. The sample was then dipped in a 5% APMES solution for 60 min to perform functionalization of the amine groups. Finally, the AuNPs were coated on the nanowires for 30 min using the Au colloid solution utilized to form the adhesion layer as shown in Fig. 1(a). At that time, the buffer solution of the Au colloid solution was replaced by deionized (DI) water because ZnO is vulnerable to acid^47^. Electrostatic adsorption is a commonly used method for attaching AuNPs on substrates in the fabrication of LSPR sensors^48–50^. Other methods are to immobilize AuNPs on the ZnO surface by covalent bonds instead of electrostatic bonds using functional groups such as thiol, cyanide, diphenylphosphine^51^. However, these methods require a few more surface treatments, which can increase the time and cost for sensor fabrication, and complicate the process.

### Experimental set-up

**Figure 2:**
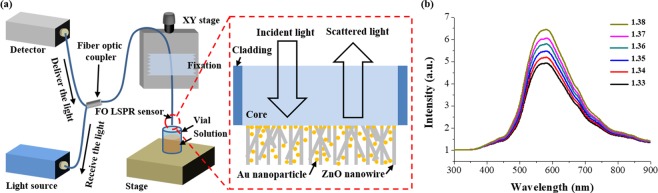
Schematic diagram of the optical system and the LSPR spectra measured using the proposed sensor: (**a**) Measurement set-up based on the optical fiber and (**b**) measured spectra via 3D FO-LSPR sensor for solutions of different refractive indices.

As shown in Fig. 2(a), a fiber-optic-based measurement set-up was configured with a light source, a detector, and a 2 × 1 optical fiber coupler (Thorlabs). For the two ends of the fiber coupler, one end was connected to the light source via the face contact/physical contact connector and the other end was connected to a detector using a subminiature version A patch cable. The other end was spliced to the fabricated FO-LSPR sensor using a fusion splicer (A-80S, Walfront, China). The sensor was fixed to an XY stage and reacted with various solutions in a vial. At this time, light from the light source is radiated to the sensor surface through total internal reflection within the core of the optical fiber. The incident light is scattered on the surface AuNPs, which is again collected by the optical fiber and transmitted to the detector through the fiber coupler.

Figure 2(b) shows the measured spectra for the FO-LSPR sensor with a 3D distribution of nanoparticles using white light (LS-1, Ocean optics, USA) and a spectrometer (SM200, Spectral Products, USA) in solutions of different refractive indices (Series AAA, Cargille Labs, USA). The proposed sensor exhibited clear LSPR spectra with intensities that increased linearly for refractive indices from 1.33 to 1.38 at intervals of 0.01. A determination coefficient of 0.996 was calculated between the increase of the intensity of the LSPR at the peak point and refractive index changes. This indicates that there is a high correlation between the two variables. A resonance peak was observed at 582 nm for a refractive index of 1.33, which is similar to the resonance wavelength of commonly known AuNPs^52^. The measurement results for the LSPR spectra confirm that the proposed FO-LSPR sensor is promising for sensitive refractive index measurements.

## Results and Discussion

### Fabrication result

**Figure 3:**
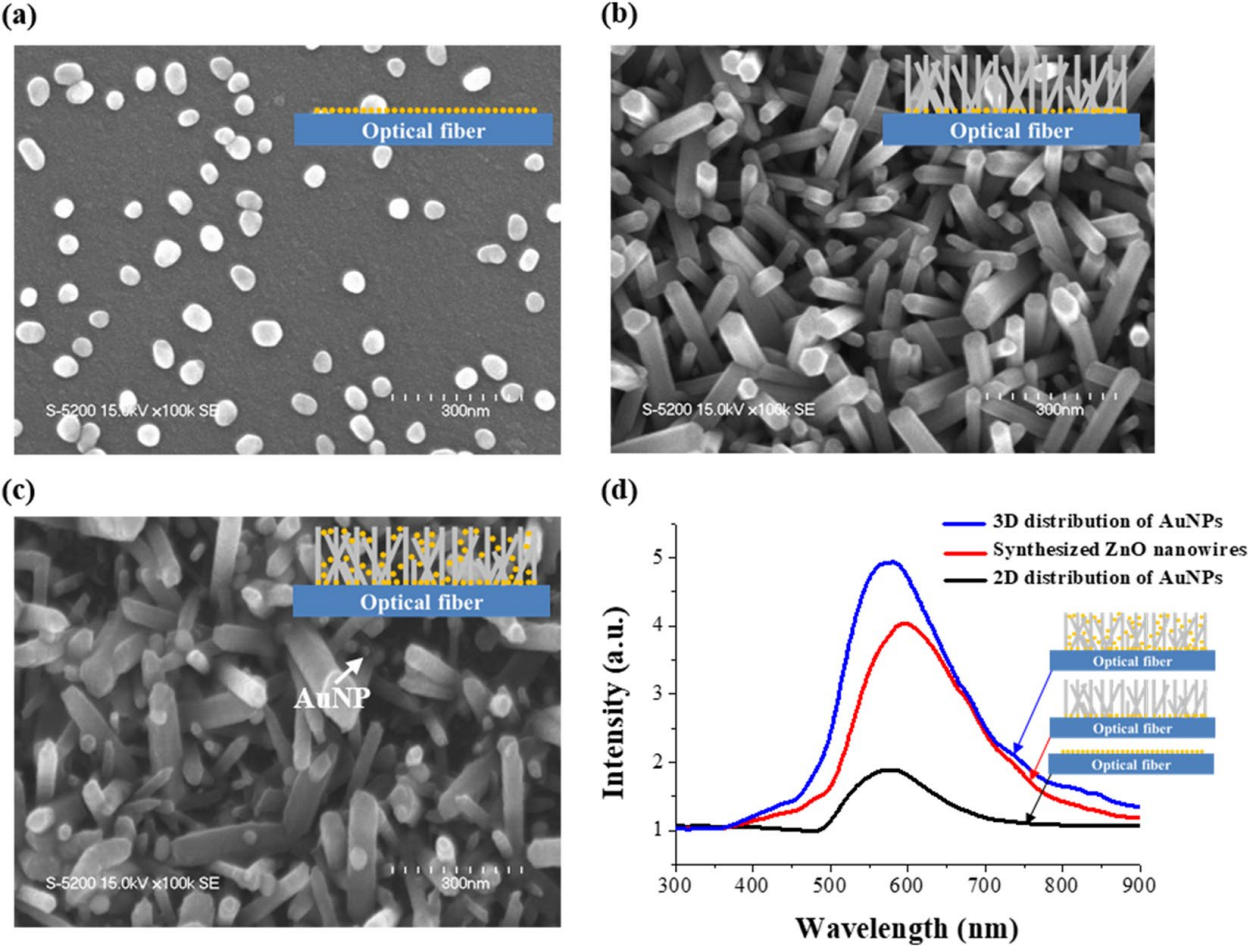
FE-SEM images of (**a**) monolayer of AuNPs on an optical fiber, (**b**) ZnO nanowires grown by AuNPs adhesion layer, (**c**) AuNPs immobilized on ZnO nanowires and (**d**) spectra for each fabrication step.

**Figure 4:**
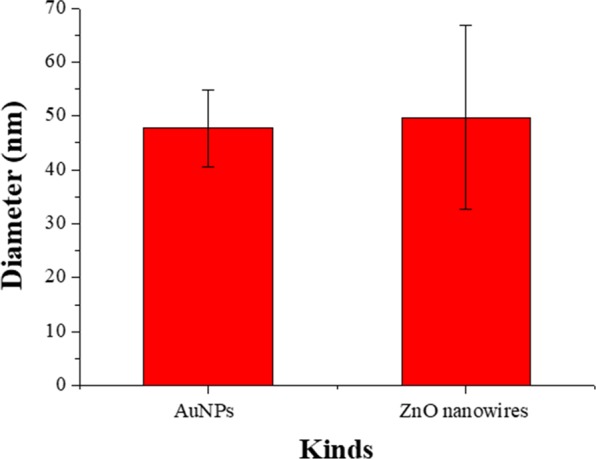
Comparison of the diameters between AuNPs and ZnO nanowires. The diameters of the AnNPs and nanowires were 47.7 ± 7.2, 49.8 ± 17.1 nm, respectively.

Figure 3 represents field emission scanning electron microscopy (FE-SEM, S-5200, Hitachi, Japan) images and the measured spectra according to each fabrication step in Fig. 1. FE-SEM images of the adhesion layer composed of monodispersed AuNPs on the surface of the optical fiber are shown in Fig. 3(a). It is evident that the AuNPs mostly exist as monomers and are evenly distributed on the fiber end-face. An image of the ZnO nanowires grown via hydrothermal synthesis is shown in Fig. 3(b), in which the well-aligned ZnO nanowires are observed with uniform length and diameter. In Fig. 3(c), the AuNPs are three-dimensionally arranged by the nanowires on the optical fiber. In each fabrication step, the spectrum was measured at a refractive index of 1.33 (Fig. 3(d)). For the spectrum shown in Fig. 3(a) due to the 2D FO-LSPR sensor, a relatively small LSPR intensity was observed because of the narrow sensing region of the AuNPs that was arranged as a monolayer on the end-facet of the optical fiber. The measured spectrum in the structure of Fig. 3(b) was slightly red-shifted when compared to the spectrum of Fig. 3(a) because the AuNPs were covered by ZnO nanowires. In the proposed structure shown in Fig. 3(c), the LSPR spectrum has an intensity that is 261% higher compared to the 2D distribution of AuNPs (Fig. 3(a)) due to the increase of the sensing area associated with the 3D distribution of nanoparticles and the light trapping effect of the nanowires. The positions of the peak resonance wavelength and the spectral shapes were almost identical for the 2D and 3D FO-LSPR sensors. This indicates that the proposed fabrication process improves the performance of an FO-LSPR sensor without degradation of the optical properties. The diameter of the AuNPs used as the adhesion layer and the diameter of the fabricated ZnO nanowires are compared in Fig. 4. The diameters of the AnNPs and ZnO nanowires were similar because the ZnO nanowires were grown on the basis of ZnO nanoparticles immobilized on the surface of AuNPs.

### Comparison of refractive index sensitivities

**Figure 5:**
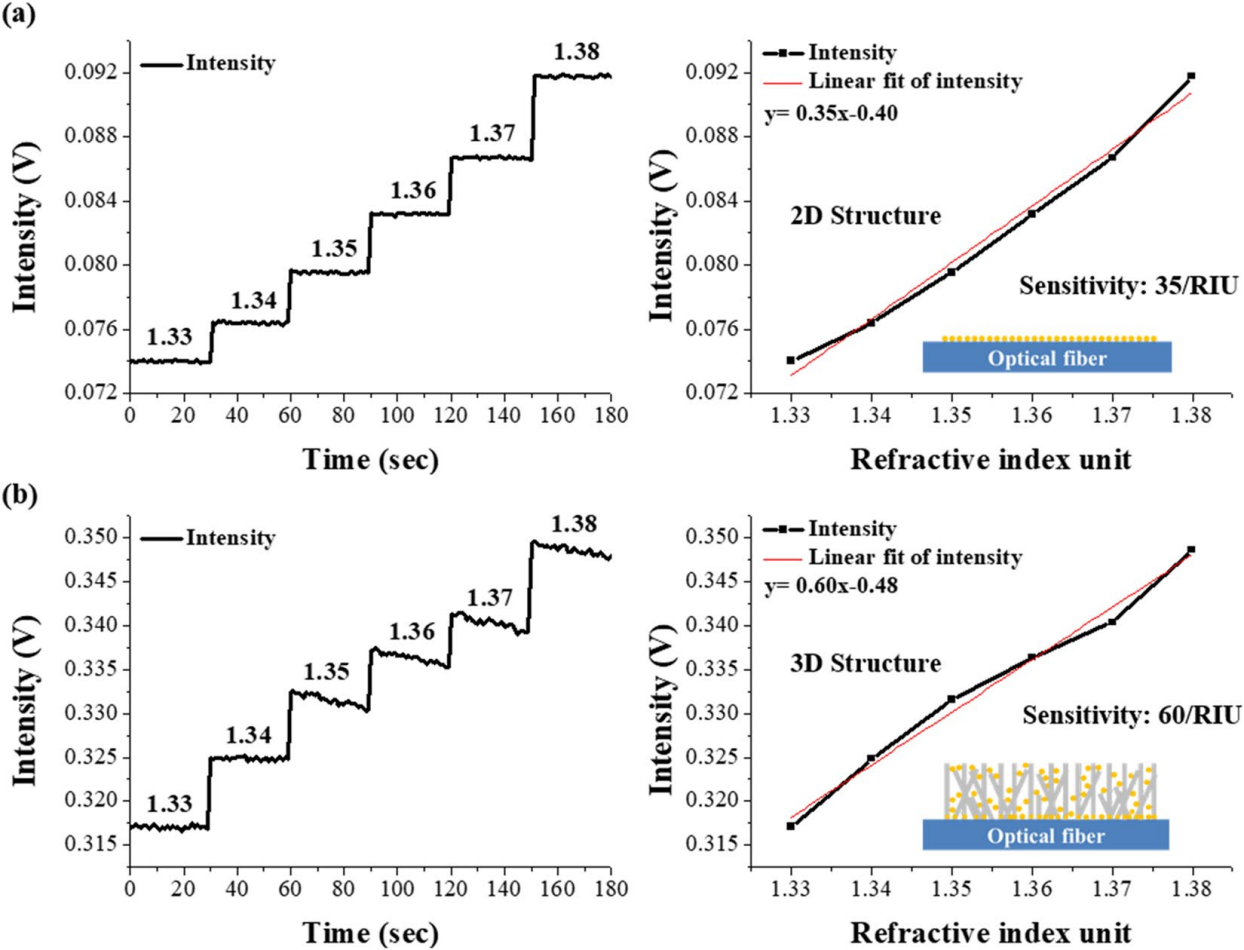
Comparison of refractive index sensitivities between (**a**) 2D structure and (**b**) 3D structure for solutions with different refractive indices.

To evaluate the enhanced sensitivity of the proposed sensor, the LSPR signal changes for solutions of various refractive indices were measured for the 2D structure (Fig. 3(a)) and the 3D structure (Fig. 3(c)). The 2D and 3D FO-LSPR sensors were sequentially immersed in solutions of different refractive indices and the LSPR intensities were observed using a laser source (Iflex-2000, Qioptiq, UK) and a photodetector (PDA36A, Thorlabs). Figure 5(a,b) represent the results measured using the 2D and 3D structures, respectively. In both sensors, the LSPR responses showed linear increases with the refractive index. For each structure, the sensitivity, which is defined as the ratio of the LSPR intensity change per unit change of the refractive index was calculated and then compared with each other. The refractive index sensitivity increased from 35 /RIU to 60 /RIU for the 3D FO-LSPR sensor, which was approximately 171% when compared to the 2D FO-LSPR sensor. Herein, RIU means refractive index unit. The figure of merit (FOM) which is defined by FOM = refractive index sensitivity/full width at half maximum (FWHM) is also calculated in 2D and 3D structures^53^, and the FOMs were 0.27 and 0.36, respectively. The FOM was improved by about 133% in the 3D distribution of AuNPs when compared to the 2D structure. The improvement of the FOM was slightly lower than the enhancement in sensitivity because of broaden FWHM in the 3D distribution of AuNPs, which was estimated due to the enhanced scale of LSPR signal with the increase of the number of nanoparticle and the partial aggregation of AuNPs between nanowires^54^. We expect that the performance of this hybrid structure can be further improved by optimizing the AuNPs density and size, in addition to changing the density and length of the ZnO nanowires.

### Biosensing performance of ZnO nanowires and AuNPs composite structure

Today prostate cancer is a major cause of death in about 10% of all cancer patients^55^. The frequency of diagnosis of prostate cancer was dramatically increased over the last few years since the prostate-specific antigen (PSA) is applied as a biomarker to detect prostate cancer. In other words, PSA is considered as the most useful biomarker for prostate cancer determination, and it is also widely used for early diagnosis and post-care of prostate cancer^56^.

PSA has a relatively small molecular weight of about 33 kDa compared to other antigens, and the reference level of PSA for the determination of prostate cancer is lower than other diseases^57,58^. In the clinic, the widely known PSA reference level is 4 ng/ml, which is lower in younger men. For these reasons, the sensor to detect PSA should have highly sensitive property. In addition, early prostate cancer may not cause any symptom and remain undetected until it is advanced, and if it is diagnosed in the early stage, prostate cancer may be more successfully treated so it is important that PSA below the reference level is measured^59^. The sensitive monitoring of PSA level is also particularly important in patients undergoing surgical prostatectomy because detecting the small changes of PSA concentration in the body is significant in the diagnosis of early recurrence of prostate cancer^60^. We performed a PSA immunoassay with various concentrations using 2D and 3D FO-LSPR sensors, respectively, and compared the results.

**Figure 6:**
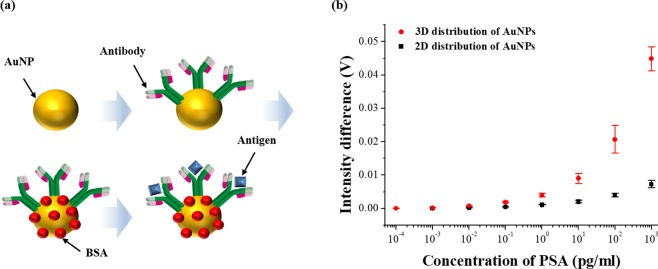
Schematic diagram of antibody-antigen interaction and measurement results in 2D and 3D structures: (**a**) a series of immunoassay protocols and (**b**) the intensity changes detected by each sensor according to various concentrations of PSA.

Figure 6(a) briefly shows the immunoassay protocol. Firstly, the sensor was immersed in a buffer solution (DI water) for 5 min to wash the sensor’s surface and stabilize the LSPR intensity. Antibodies were then absorbed on the AuNPs for 15 min in 20 µg/ml PSA antibody (PSA 10, Fujirebio, Japan) in borate buffer (0.05 M, pH 8.5, Bioworld, USA). After the unattached antibodies were washed with buffer solution, the sensor was incubated in 1% bovine serum albumin (BSA, 98%, Sigma Aldrich) aqueous solution for 15 min. BSAs immobilized on AuNPs prevent non-specific binding except for antibody-antigen interaction by blocking the area where the antibody was not fixed^61^. The sensor was then rinsed in buffer solution and then reacted with 100 pg/ml PSA solution to an induce antibody-antigen reaction. Finally, the sensor’s surface was washed again using a buffer solution to flush the unbound antigens and the net changes of the LSPR intensities were recorded using a photodetector in the buffer solutions before and after the antigen reaction.

In Fig. 6(b), the 2D and 3D FO-LSPR sensors were applied to measure the differences between the intensities before and after the antibody-antigen reaction in various levels of PSA. Each concentration was measured three times using different sensor and the averages and standard deviations measured by 2D and 3D sensors were shown in Fig. 6. In each sensor, the limits of detection (LODs) were calculated based on the definition of LOD = 3 × (the standard deviations of the intensity differences)/the slope of the fitting line^62^. The concentration of which the mean of measured values was detected more than three times the standard deviation was decided as the lowest concentration that is measurable by the proposed sensor^63^. In Fig. 6(b), the range of measurable concentrations was 0.01 pg/ml–1 ng/ml and 0.001 pg/ml–1 ng/ml in the 2D and 3D FO-LSPR sensors, respectively. The averages of the measured values at 0.001 pg/ml with 2D sensor and at 0.0001 pg/ml with 3D sensor were less than three times the standard deviations. The measurement range was enhanced in 3D structure because of relatively bigger LSPR intensities. The LODs were calculated based on the slope which is fitted with linear function by the averages of intensity differences in measurable range. The LODs in 2D and 3D FO-LSPR sensors were determined to be 2.06 and 0.51 pg/ml, respectively. The LOD was improved by 404% in the 3D distribution of nanoparticles when compared to the 2D distribution of nanoparticles. In addition, the selectivity of proposed sensor was evaluated in Fig. S3, and the coefficients of variation (CVs) were calculated in the range of measurable concentrations as an indicator of the reproducibility of the measured values. The CV is converted to a percentage by dividing the standard deviation of the measured values by the mean. The means of CVs in the 2D and 3D sensors were 14.1 and 20.3%, respectively. The increased CV in the 3D FO-LSPR sensor was assumed to be due to the added process to fabricate the 3D structure. Despite the increase of CV in the 3D FO-LSPR sensor, CV around 20% is good to be applied to immunoassay^64^. This can be improved through optimization of growth conditions of zinc oxide nanowires and immobilization conditions of Au nanoparticles. In other researches where sensors based on the surface enhanced Raman scattering, colorimetric, surface plasmon resonance, and fluorescence were used for the measurement of PSA, the LOD were 5, 20, 91, 200 pg/ml, respectively^65–68^. On the other hand, the LOD of the proposed sensor was 0.51 pg.ml, which is about 10–100 times lower when compared with other researches. These results guarantee that the 3D FO-LSPR sensor is suitable for general applications in the biosensing field that the early detection of diseases and recurrences is required.

## Conclusions

To improve the sensitivity of optical fiber based LSPR sensor, 3D nanowires were grown on the surface of an optical fiber having compactness, portability, and ease of handling and AuNPs were immobilized between the nanowires. A 3D support based on nanowires increases the number of AuNPs per unit plane with an enhanced surface area and focuses light on the sensing region due to its forest-like structural characteristics. The porous structure of nanowires also creates an environment in which the target molecules can easily access the surface of the AuNPs. This concept was demonstrated by experimenting with a sensor with a 3D distribution of nanoparticles and a superior performance was observed compared to an FO-LSPR sensor with a 2D array of nanoparticles. When the refractive index sensitivity of 2D and 3D FO-LSPR sensors was compared, the sensor based on the ZnO nanowires deposited with AuNPs showed an improved sensitivity of approximately 171%. In addition, each sensor was utilized to detect the PSA with various levels, and the LODs were 2.06 and 0.51 pg/ml, respectively. The 3D FO-LSPR sensor identified the antigen with a higher sensitivity of 404%. As a result, the FO-LSPR sensor with a 3D structure that utilizes both nanowires and AuNPs is expected to have broad applications in high sensitivity real-time label-free biosensors.

## Supplementary information

SUPPLEMENTARY MATERIALS
