# Supplementary material for: Fiber optic sensor based on ZnO nanowires decorated by Au nanoparticles for improved plasmonic biosensor
Source: Sci Rep. 2019 Oct 30;9:15605. doi: 10.1038/s41598-019-52056-1 (PMC6821738; doi:10.1038/s41598-019-52056-1)
Supplement: Supplementary file 1 — SUPPLEMENTARY MATERIALS [file 41598_2019_52056_MOESM1_ESM.docx]

**Supplementary information**

**Fiber optic sensor based on ZnO nanowires decorated by Au nanoparticles for improved plasmonic biosensor**

Hyeong-Min Kim, Jae-Hyoung Park^*^ & Seung-Ki Lee^*^

Department of Electronics and Electrical Engineering, Dankook University, Yongin 16890, South Korea

*Correspondence: [parkjae@dankook.ac.kr](mailto:parkjae@dankook.ac.kr), [skilee@dankook.ac.kr](mailto:skilee@dankook.ac.kr)

**List of contents**

1. UV-Vis spectrum of synthesized Au nanoparticles
2. X-ray diffraction pattern of grown ZnO nanowires
3. Selectivity of the device
4. **UV-Vis spectrum of synthesized Au nanoparticles**

**Figure S1.** UV-Vis spectrum of synthesized Au nanoparticles.

The bottom-up method that reduces the Au ions into nanoparticles can cheaply synthesize a large number of Au nanoparticles. The most typical method is to reduce the HAuCl4 aqueous solution with sodium citrate aqueous solution as a reduction agent, and it is known that spherical Au nanoparticles are formed. To synthesize the Au nanoparticles, 250 µM Au (Ⅲ) chloride trihydrate aqueous solution of 50 ml was heated in a silicon oil bath until the temperature reached 100℃. Then 0.5 ml of 35 mM sodium citrate dehydrate aqueous solution was added and vigorously stirred for 20 min. At this time, the color of the solution was changed from bright yellow to ruby red. The nanoparticles of various sizes can be synthesized by controlling the amount of sodium citrate dehydrate aqueous solution. In this paper, the average size and standard deviation of the fabricated nanoparticles were 47.7±7.2 nm. In addition, the prepared Au nanoparticles were analyzed by UV-Vis spectrometer (UV1800, Shimadzu, Japan). In Fig. S1, a resonance peak was observed at 555 nm, which is to the resonance wavelength of commonly known Au nanoparticles.

1. **X-ray diffraction pattern of grown ZnO nanowires**


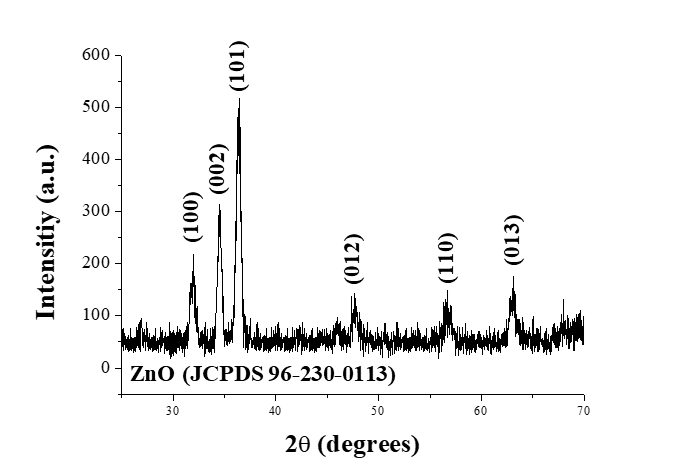


**Figure S2.** XRD patterns of ZnO nanowires fabricated by hydrothermal synthesis.

In order to characterize the crystal structure of ZnO nanowires grown by hydrothermal synthesis, X-ray diffraction (XRD, Ultima Ⅳ, Rigaku Corporation, Japan) patterns were measured and identified using Cu-Kα radiation at a scan rate of 5°/min. Data in Fig. S2 was taken for the 2θ range from 25° to 70°. As a result, six peaks at 2θ values of 31.76, 34.73, 36.28, 47.70, 56.55, and 63.12° corresponding to (100), (002), (101), (012), (110), and (013) plane of ZnO nanowires were observed and compared with the joint committee on powder diffraction standards (JCPDS) card. The XRD patterns confirm that the resultant structures are ZnO nanowires. We expect that the alignment of ZnO nanowires can be further improved by optimizing the nanowires growth conditions such as the molarity of growth solution, the growth time and temperature.

1. **Selectivity of the device**

**Figure S3.** The nonspecific binding measurement between the proposed sensor with PSA antibodies and another biomarker (AFP) of varying concentrations.

To confirm the specific binding of the proposed sensor to prostate-specific antigen (PSA), nonspecific binding between the sensor to which the PSA antibody is immobilized and another biomarker experimented. Alpha-fetoprotein (AFP) which is related to liver cancer was reacted at concentrations similar to the range of PSA measurement by our sensor. In Fig. S3, each concentration was measured three times using the prepared fiber-optic-based localized surface plasmon resonance sensors. As a result, the sensor to which the PSA antibody was fixed outputted insignificant intensity differences when reacted with various levels of AFP because of low specific binding to AFP except for PSA. AFP has a molecular mass of about 70 kDa, which is more than twice the molecular mass of a PSA with a 33 kDa. Nevertheless, meaningless signal changes indicate that the proposed sensor can be used as cancer detectors to selective biomarker measurement by introducing appropriate receptors.
